# Editorial: Algal genetics and genetic Breeding

**DOI:** 10.3389/fgene.2022.1019621

**Published:** 2022-10-31

**Authors:** Zhenghong Sui, Naihao Ye, Tifeng Shan

**Affiliations:** ^1^ Key Laboratory of Marine Genetics and Breeding (Ocean University of China), Ministry of Education, Qingdao, China; ^2^ Yellow Sea Fisheries Research Institute, Chinese Academy of Fishery Sciences, Qingdao, China; ^3^ Function Laboratory for Marine Fisheries Science and Food Production Processes, Qingdao National Laboratory for Marine Science and Technology, Qingdao, China; ^4^ CAS and Shandong Province Key Laboratory of Experimental Marine Biology, Center for Ocean Mega-Science, Institute of Oceanology, Chinese Academy of Sciences, Qingdao, China

**Keywords:** algae, genetics, genetic breeding, high throughput, genome scale

Algae are the main source of primary productivity in water systems and an important economic resource due to the various products they provide. According to a report on *The State of World Fisheries and Aquaculture*, fisheries and aquaculture production reached a record high of 214 million tonnes, among which algae (including both micro and macro) accounted for 36 million tonnes in 2020 (FAO 2022).

During the past few decades, there has been rapid progress in biological technology, especially molecular biological techniques, equipping us with many powerful tools such as high-throughput sequencing and genome-scale analysis to promote our knowledge and applications of algal genetics and breeding.

This Research Topic aimed to collect contributions of high-quality research on algal genetics and breeding, such as genetic dissection *via* high-throughput means; development of breeding approaches with high efficiency; germplasm innovation applications with genome-scale tools; genomics, and proteomics; as well as information about the establishment and application of genome editing technologies.

In one study, Lin et al. selected two strains of *Pyropia haitanensis* that show significant differences in conchosporangia maturation for high-throughput sequencing analysis. The key molecular pathways and genes involved in conchosporangia maturation were identified through weighted gene co-expression network analysis (WGCNA). Phosphorus metabolic processes, lipid metabolism, and the PI signaling systems were identified as being important metabolic pathways during conchosporangia maturation in *P. haitanensis*. The hub genes that positively responded during conchosporangia maturation were diacylglycerol kinase (DGK) and phosphatidylinositol-3-phosphate-5-kinase (PIKFYVE), which are involved in the synthesis of phosphatidic acid (PA), a key component of lipid metabolism. A full-length DGK sequence of *P. haitanensis*, designated as PhDGK1, was obtained and conserved motif and phylogenetic tree analyses showed that PhDGK1 belongs to DGK Cluster II. The transcriptional analysis of PhDGK1 suggested that it not only participates in the formation of conchosporangia but also affects the speed of conchosporangia maturation. Lipid metabolism may play a key role in regulating conchosporangia maturation in *Pyropia* spp.

Another study in the Research Topic by Kim et al. investigated the influence of geographic isolation on the genomic and genetic diversity of two of the most widespread worldwide species, *A. plicata* and *A. fastigiata*, in an agarophyte *Ahnfeltia* (Ahnfeltiales, Rhodophyta). New plastid (ptDNAs) and mitochondrial genomes (mtDNAs) were assembled for these *Ahnfeltia* species from four different regions (*A. plicata*–Chile and United Kingdom and *A. fastigiata*–Korea and Oregon). Two architecture variations were identified in *Ahnfeltia*: in ptDNA of *A. fastigiata* Oregon, the hypothetical pseudogene region was close to the *Agarophyton* plasmid sequence, and may be translocated, likely *via* recombination with palindromic repeats or by undertaking a gene transfer event from the plasmid. The composition of the group II intronic ORFs in the mtDNA of *A. fastigiata* Korea was distinct from others, indicating that it experienced special events involving the gain and loss of group II intronic ORFs. The overall gene contents of the organelle genomes of *Ahnfeltia* were conserved. Monophyly was supported in Ahnfeltiophycidae by phylogenetic analysis using concatenated genes from ptDNAs and mtDNAs. The most probable individual gene trees showed that the *Ahnfeltia* populations were genetically diversified. The cox1 haplotype network as well as a dN/dS analysis all provide evidence as to the consistency between genetic diversity and geographic distribution within *Ahnfeltia* populations. Overall, the study suggests that geographic distribution has affected the genomic and genetic characteristics of *Ahnfeltia*.

Another study by Shan and Pang reviewed *Undaria pinnatifida*, a commercially important brown alga, whose global annual yield has been more than two million tonnes since 2012. It is extensively cultivated in East Asia and is mainly used as food as well as feed for aquacultural animals, or as raw materials for the extraction of chemicals in pharmaceutics and cosmetics. During cultivar breeding, it is vital to know every detail of the life history. The cultivar breeding and cultivation methods of *U. pinnatifida* have usually been learned or are directly transferred from those of *Saccharina japonica,* because they share common basic life history. However, recent studies have revealed certain peculiarities in the life history of *U. pinnatifida*. In this article, the following parts relevant to cultivar breeding in this alga were included: 1) the peculiar component of the life history; 2) the genetics, transcriptomics, and genomics tools available; 3) the main cultivar breeding methods. The authors also propose a prospect for the establishment of genetic editing system in *U. pinnatifida.*

A study by Zeng et al. researched myeloblastosis (MYB) proteins, which are one of the largest families of transcription factors (TFs) found in nearly all eukaryotic organisms, regulating important processes in growth and development. There are studies on MYBs from animals and plants; however, comprehensive analysis of SAR (stramenopiles, alveolates, and rhizarians) is limited. In this study, the structure, evolution, and expression of MYBs in four brown algae comprising the biggest multicellular lineage of SAR were characterized. A total of 172 MYB genes were identified. Subfamily 1R-MYB displayed fewer conserved motifs outside the MYB domain, comprising heterogeneous proteins and the largest subgroup of brown algal 1R-MYBs. While there are more 2R-MYBs members in plants, brown algae had more 3R-MYBs than 2R-MYBs, displaying 3R-MYB expansion in the common ancestor of brown algae. Brown algal MYBs were found with ancient origins and a diverged evolution as revealed by phylogenetic analysis, which was close to stramenopile species, compared with their relationship with red algae, green algae, or animals, suggesting that brown algal MYBs did not come from the secondary endosymbiosis of red and green plastids. Sequence comparison revealed that the repeat of 1R-MYBs was similar to the R3 of 2R and 3R-MYBs, which suggested that 1R-MYB was derived from the loss of the first and second repeats of the ancestor MYB. Brown algal MYB exhibited a higher proportion of intrinsically disordered regions compared with other species of SAR, which might contribute to multicellular evolution. Expression analysis confirmed the involvement of many MYB genes in stress response and developmental control.
